# Case of Human Orthohantavirus Infection, Michigan, USA, 2021

**DOI:** 10.3201/eid3004.231138

**Published:** 2024-04

**Authors:** Samuel M. Goodfellow, Robert A. Nofchissey, Dustin Arsnoe, Chunyan Ye, Seonghyeon Lee, Jieun Park, Won-Keun Kim, Kartik Chandran, Shannon L.M. Whitmer, John D. Klena, Jonathan W. Dyal, Trevor Shoemaker, Diana Riner, Mary Grace Stobierski, Kimberly Signs, Steven B. Bradfute

**Affiliations:** University of New Mexico Health Sciences Center, Albuquerque, New Mexico, USA (S.M. Goodfellow, R.A. Nofchissey, C. Ye, S.B. Bradfute);; US Department of Agriculture Animal and Plant Health Inspection Service Wildlife Services–Michigan Program, Okemos, Michigan, USA (D. Arsnoe);; Hallym University College of Medicine, Chuncheon, South Korea (S. Lee, J. Park, W.-K. Kim);; Albert Einstein College of Medicine, Bronx, New York, USA (K. Chandran);; Centers for Disease Control and Prevention, Atlanta, Georgia, USA (S.L.M. Whitmer, J.D. Klena, J.W. Dyal, T. Shoemaker);; Michigan Department of Health and Human Services, Lansing, Michigan, USA (D. Riner, M.G. Stobierski, K. Signs)

**Keywords:** Orthohantavirus, bunyavirdiae, zoonoses, infection, genetics, transmission, reservoir, Michigan, United States, viruses, Sin Nombre virus, New York virus

## Abstract

Orthohantaviruses cause hantavirus cardiopulmonary syndrome; most cases occur in the southwest region of the United States. We discuss a clinical case of orthohantavirus infection in a 65-year-old woman in Michigan and the phylogeographic link of partial viral fragments from the patient and rodents captured near the presumed site of infection.

Orthohantaviruses are negative-sense, enveloped RNA viruses that are transmitted by host reservoirs, such as rodents, to humans. Human infection occurs through inhalation of aerosolized viral particles from host excreta, such as urine or feces, often in enclosed spaces during infestations. New World orthohantavirus infection results in hantavirus cardiopulmonary syndrome (HCPS), which consists of febrile illness with edema and respiratory failure (*1*). In the United States, most HCPS cases occur in the Southwest and have a ≈35% mortality rate (*2*).

The dominant orthohantavirus that causes HCPS in the United States is Sin Nombre virus (SNV), which is thought to be carried and transmitted by the western deer mouse (*Peromyscus sonoriensis*). New York virus (NYV) is another pathogenic variant of orthohantavirus that is found in white-footed deer mice (*Peromyscus leucopus*); cases occur primarily in the Northeast region of the country (*3*). Although multiple host reservoirs for orthohantaviruses are distributed throughout the United States, most human cases are caused by SNV (*4*,*5*).

In early May 2021, a previously healthy 65-year-old woman visited an emergency department in Washtenaw County, Michigan, USA, with febrile prodrome of 3–6 days, thrombocytopenia, mild transaminase elevation, and acute hypoxic respiratory failure of unclear etiology requiring intubation. An extensive infectious disease workup was conducted, and physicians initially ruled out such pulmonary pathogens as SARS-CoV-2, common respiratory viruses, fungal agents, and *Legionella* spp. The family was interviewed to obtain a travel and animal exposure history, which revealed that the patient had not traveled outside of Michigan in the previous year. The interview also confirmed that the patient had not consumed unpasteurized dairy or undercooked meat, had a mostly indoor dog, lived near a natural area but used trails/sidewalks, and had no known rodent infestation in the home. However, the spouse reported that the patient had spent time recently cleaning out a relative’s home that had been uninhabited for 2 years and was infested with mice. 

Results of a tickborne disease panel were negative, but hantavirus antibody testing performed at a commercial lab showed positive results for both IgM and IgG. The treating hospital notified the Michigan Department of Health and Human Services of a case of HCPS. Confirmatory hantavirus testing was arranged and confirmed with the Centers for Disease Control and Prevention, using serum samples collected from hospitalization.

Trapping was performed in and around the suspected site of exposure (relative’s home) using Sherman folding traps (https://shermantraps.com; 94 trap nights), resulting in 12 rodents captured (12.8% trap success) under an approved animal-use protocol (*6*). Trapping was conducted 12 days after the patient was released from the hospital. Researchers observed signs of previous trapping efforts; 5 unusable *Peromyscus* mouse carcasses were found in snap traps in the residential basement. Signs of infestation were evident. Of the 12 trapped rodents, 3 (25%) were *P. leucopus* mice, 1 (8%) was a Northern short-tailed shrew (*Blarina brevicauda*), and 8 (67%) were Eastern chipmunks (*Tamias striatus*) (Table). The surrounding flora consisted of lawns, shrubs, and an evergreen windbreak near a public trail.

**Table:** Measurements, location, and quantitative PCR results from captured rodents at likely site of patient orthohantavirus exposure, Michigan, USA, 2021*

Sample ID	Species (common name)	Weight, g	Total length, mm	Tail length, mm	Hind foot length, mm	Ear size, mm	Age/sex	Location of capture	PCR+ tissue (Ct values)
YR-01	*Peromyscus leucopus *(white-footed mouse)	13.2	152	75	19.5	16.5	Subadult/M	Garage right front corner	Kidney (33, 33), BAF (35, 35)
YR-02	*Blarina brevicauda* (Northern short-tailed shrew)	18	115	27	15	2.5	Adult/F	Backyard	NA
YR-03	*P. leucopus*	21	174	86	20.5	17.5	Adult/F	Backyard	Kidney (39, 39), liver (38, 34)
YR-04	*Tamias striatus* (Eastern chipmunk)	84.5	221	75	33.5	18	Adult/F	Backyard	NA
YR-05	*T. striatus*	73.5	222	85	36.5	18	Adult/M	Backyard	NA
YR-06	*P. leucopus*	17	159	73	20.5	17	Subadult/M	Porch right back corner	NA
YR-07	*T. striatus*	90	225	83	35	14.5	Adult/F	Neighbor backyard, right side	NA
YR-08	*T. striatus*	91	224	83	35	19	Adult/F	Neighbor backyard, right side	NA
YR-09	*T. striatus*	88	227	93	34	15	Adult/F	Neighbor backyard, right side	NA
YR-10	*T. striatus*	94	202†	48†	36	18	Adult/M	Backyard	Lung (35, 35)
YR-11	*T. striatus*	58	214	85	36	14	Subadult/M	Backyard	NA
YR-12	*T. striatus*	49	196	72	37	17	Subadult/M	Backyard	Lung (39, 39)

*BAF, brown adipose fat; Ct, cycle threshold; ID, identification; PCR+, positive result determined by quantitative reverse transcription PCR. †Bobbed tail.

Using quantitative reverse transcription PCR, we screened lung, liver, brown fat, or kidney tissue from captured rodents and from a plasma sample of the patient obtained during hospitalization (*6*). Brown fat and kidney tissue from 2 *P. leucopus* mice and lung tissue from 2 *T. striatus* chipmunks tested positive for SNV. Three fragments were obtained from the patient sample, 1 for the short segment (480 bp), 1 for the medium segment (283 bp), and 1 for the large segment (377 bp). Similar fragments were also generated from 3 of the 4 infected rodents; all sequences are publicly available in GenBank (accession nos. OR428177–88). We compared fragments by using phylogenetic analysis against several known orthohantavirus reference sequences to determine potential identification. The partial sequences of SNV short and medium segments from the patient formed a phylogenetic lineage with SNV sequences from the rodents collected in or near the suspected site of exposure in Michigan. However, the patient’s large fragment formed a lineage with NYV, suggesting that this species may be an SNV or NYV variant (Figure).

**Figure:**
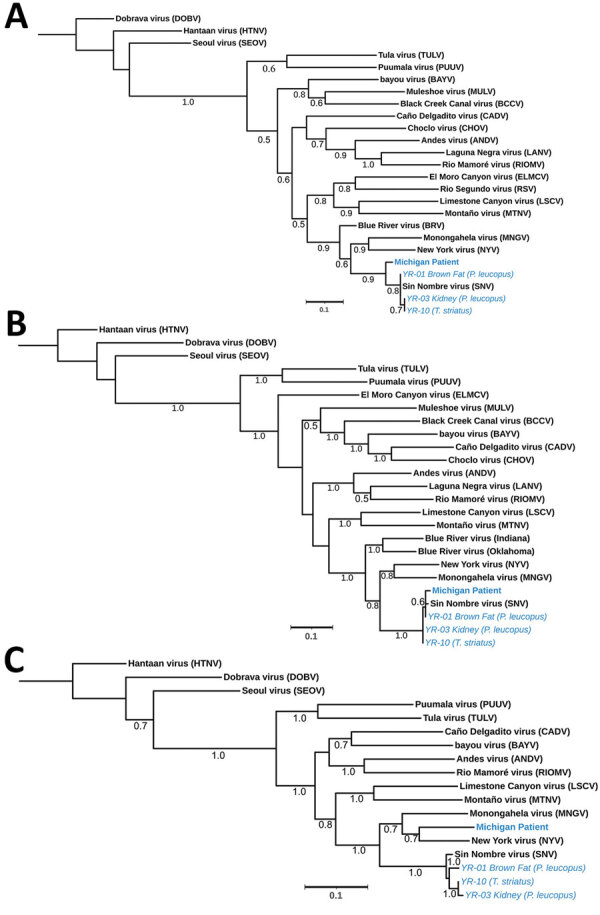
Phylogenetic analysis of orthohantavirus sequence fragments from samples taken from a 65-year-old woman in Michigan, USA, and trapped rodents from the likely site of exposure (blue text). Trees displaying the patient small fragment (481 bp) (A), medium fragment (283 bp) (B), and large fragment (377 bp) (C) were aligned against wild-caught rodents near site of exposure and reference sequences. Numbers along branches indicate bootstrap values of 500 replicates. GenBank accession numbers: human patient, OR428177–9; YR-01, brown adipose fat from a *Peromyscus leucopus* white-footed mouse, OR428180–2; YR-03, kidney tissue from a *P. leucopus* mouse, OR428183–5; and YR-10, lung tissue from a *Tamias striatus* Eastern chipmunk, OR428186–8. Scale bars indicate number of substitutions per site.

Previously, we identified the likely site of rodent-to-human SNV transmission in a patient case study (*6*). Here, we attempted a similar approach but were only able to generate partial sequences for the patient sample, which we compared with captured rodents. Orthohantavirus incubation periods can be up to several weeks after exposure (*7*), which may impact the timeliness of trapping efforts. We found infected *P. leucopus* mice and *T. striatus* chipmunks at the site of exposure, both of which have been reported to carry NYV or SNV; *P. leucopus* mice are susceptible and capable vessels for SNV replication after laboratory infection (*6**,**8**–**10*). This finding suggests that orthohantaviruses may not be as species host–restricted as previously thought. Further studies are warranted to clarify (or define) orthohantavirus species in Michigan to anticipate the risk for patient infection. Increasing surveillance and diagnostic efforts can enable prospective detection of circulating viruses. 
